# Poor, old and in need of care: A qualitative study about the consequences for home care and participation

**DOI:** 10.3205/000274

**Published:** 2019-08-09

**Authors:** Melanie Messer

**Affiliations:** 1 APOLLON Hochschule der Gesundheitswirtschaft, Bremen, Germany

**Keywords:** home care, poverty, patient participation, aged, multimorbidity

## Abstract

**Background:** For people in old age and in need of care, there is an increased risk of being affected by poverty. The aim of the study was to explore the forms of poverty that nurses in Germany perceive in older people in need of care who are living at home, as well as the perceived strategies to deal with this situation regarding health and nursing care and the consequences for the possibilities of participation.

**Methods:** A qualitative study was performed as a secondary data analysis of 39 transcribed problem-oriented expert interviews. The data analysis was conducted through content and thematic analysis.

**Results:** Two forms of poverty among people in need of care were described by the nurses interviewed: 1) a self-imposed austerity, and 2) a material and financial poverty. The possible consequences of poverty reported in those in need of care are harmful self-restrictions and limited opportunities for participation, e.g. through the multiple use of medical aids and reduced intake of medications, but also restricted food intake. This situation is often accepted without complaint by people in need of care. However, restrictions in social activities can be a considerable burden on the quality of life of patients. The interviewees described that offering professional help can be challenging, because people in need of care and affected by poverty often show a degree of high shame in accepting financial support or negate their own precarious life situation, but also because support structures in the social and health system are limited.

**Conclusion:** The results indicate how older people in need of care impose self-restrictions due to their poverty, which are harmful to health and limit the quality of life. Furthermore, the described strategies can pose a significant risk to the medical safety of patients. This has implications for the nursing and social care, but also for the medical treatment of the affected patients. The existing support systems seem to be only partially prepared for dealing with such situations. In view of a trend towards an older population structure, future research should put more emphasis on the user-friendly adaptation of the social and health systems. Strategies to prevent and deal with poverty in older people in need of care, easy access to social support systems, as well as the preparation of the health professions for the associated tasks seem to be particularly indicated.

## Zusammenfassung

**Hintergrund:** Im höheren Lebensalter und bei Pflegebedürftigkeit besteht ein erhöhtes Risiko, von Armut betroffen zu sein. Die Studie geht der Frage nach, welche Formen von Armut beruflich Pflegende in Deutschland bei älteren Patientinnen und Patienten in der häuslichen Versorgung wahrnehmen. Zudem wird untersucht, welche Strategien im Umgang mit dieser Situation in Bezug auf die gesundheitliche und pflegerische Versorgung erlebt werden und welche Folgen dies für die Partizipationsmöglichkeiten hat.

**Methode:** Es wurde eine Sekundärdatenanalyse von 39 transkribierten problemzentrierten Experteninterviews durchgeführt. Die qualitative Datenanalyse erfolgte mittels Inhaltsanalyse und thematischer Analyse.

**Ergebnisse:** Es werden zwei Formen der Armut bei pflegebedürftigen Menschen von den interviewten Pflegenden beschrieben: 1) eine selbst auferlegte Sparsamkeit sowie 2) eine materielle und finanzielle Armut. Die möglichen Folgen, die beschrieben werden, sind schädliche Selbstbeschränkungen und eingeschränkte Partizipationsmöglichkeiten, z.B. durch den Mehrfachgebrauch medizinischer Hilfsmittel und die beschränkte Einnahme von Medikamenten, aber auch eine reduzierte Nahrungsaufnahme. Diese Situation wird von pflegebedürftigen Menschen oft ohne Beschwerden hingenommen. Einschränkungen im sozialen Bereich können hingegen eine erhebliche Belastung für die Lebensqualität der Patientinnen und Patienten darstellen. Die Interviewten beschreiben, dass das Angebot einer professionellen Unterstützung herausfordernd sein kann, da von Armut betroffene pflegebedürftige Menschen oftmals eine hohe Scham haben, finanzielle Hilfeleistungen zu akzeptieren oder ihre eigene prekäre Lebenssituation negieren, aber auch aufgrund begrenzter Unterstützungsstrukturen im Sozial- und Gesundheitssystem.

**Schlussfolgerung:** Die Ergebnisse weisen darauf hin, in welcher Weise pflegebedürftige ältere Menschen sich aufgrund ihrer Armut Selbstbeschränkungen auferlegen, die gesundheitsschädigend sind und die Lebensqualität der Betroffenen beschränken. Zudem können die beschriebenen Strategien ein erhebliches Risiko für die medizinische Sicherheit der Patienten darstellen. Dies hat Auswirkungen auf die Pflege und Sozialfürsorge, aber auch auf die medizinische Versorgung der betroffenen Patienten. Die bisherigen Unterstützungssysteme scheinen jedoch nur bedingt auf den Umgang mit solchen Situationen vorbereitet zu sein. Angesichts des Trends zu einer älteren Bevölkerungsstruktur sollte die zukünftige Forschung mehr Gewicht auf die benutzerfreundliche Anpassung der Sozial- und Gesundheitssysteme legen. Strategien zur Prävention und Bekämpfung der Armut bei pflegebedürftigen, älteren Menschen, der unkomplizierte Zugang zu sozialen Unterstützungssystemen sowie die Vorbereitung der Gesundheitsberufe auf die damit verbundenen Aufgaben scheinen hierfür mögliche Ansatzpunkte zu sein.

## Introduction

The European population is being affected by a transition towards an older population structure which is associated with an increasing proportion of people in need of care [1], [2]. Today, 19.2% of the European population is aged 65 or older [3]. There is a tendency for a steady increase, with an estimated percentage of 29.1% in 2080 [3]. In some European countries, securing the livelihood of people in this phase of life seems to be particularly challenging. The risk of poverty or social exclusion in people of 65 years and older ranges from 6.1% in the Netherlands to 51.8% in Bulgaria [4]. Where care is needed, poverty and material exclusion in old age are considered as particularly prevalent in some European countries [5], [6]. Both are associated with more frequent social deprivation, lower chance of participation and impaired quality of life – for those in need of care, but also for the caring relatives [5], [6]. Furthermore, the risk of entering need of care is associated with low socio-economic status and type of employment during working life [7].

Germany, which is Europe’s largest economy, is affected by these developments as well. With 21.2% of the total population aged 65 or older, Germany is one of the European countries with the highest proportion of older people [3]. Depending on various factors such as age, insurance period and income, most citizens in Germany are entitled to a statutory pension. However, the risk of poverty or social exclusion in Germany is faced by 18.3% of people over 65 years of age [4], [8]. Older women are of particular concern due to their employment biographies [9]. For example, 57.5% of women over 65 years of age in former West Germany receive a statutory pension of less than 600€ per month versus 28.2% of men in the same age group (at-risk-of-poverty threshold: 1,033€ per month) [10]. Where nursing care is needed, the statutory nursing care insurance in Germany provides basic support, which covers costs up to a certain amount [2]. As in other European countries, the most common form of care is domestic care: almost three quarters of the 2.9 million people in need of care in Germany are cared for at home [1], [2], [11]. Nonetheless, in the case of need for care, the risk of poverty in old age also increases here [12]. Despite these findings, there has been little research into how the people “behind the numbers” deal with their situation. Study results suggest that people in need of care with limited financial means are more likely to refrain as long as possible from professional help in order to save money [13]. Furthermore, older people in need of care with limited financial means are interested in maintaining their self-determination and their opportunities for participation [14], [15]. It can be assumed that these requirements may also become visible when nurses from a home care service are involved in the care of the older people [14]. This became apparent in a qualitative study on participation in home care from the perspective of nurses, in which patients’ financial means were repeatedly mentioned as crucial for the provision of opportunities for participation [16]. Against this background, a study with a secondary analysis of the data was conducted. The study focuses on three research questions:

1. Which forms of poverty do nurses in home care perceive among older patients with limited financial means?
2. Which strategies do nurses in home care experience among patients with limited financial means in dealing with their health and nursing care?
3. What are the consequences of limited financial means for the participation possibilities for older patients in home care?

## Methods

### Study design and ethics

The study emerged from a secondary data analysis of 39 transcribed anonymized problem-oriented expert interviews in Germany [17]. All interviews of the primary study were included. The interviews were originally collected for a qualitative study focusing on patient participation in those in home care with multimorbidity without dementia [16]. For the study, the Ethics Committee Münster, Germany considered an ethics vote after approval as not necessary. Only interviewees who gave written consent for the use of their materials for research have been included in the analysis.

### Participants and data collection

The interviews were conducted with 39 nurses (women: n=35, men: n=4) who work in home care as nursing service managers (n=17), quality managers (n=5) or nursing staff (n=17). The nurses should have completed at least three years of nursing training or academic nursing studies. Following theoretical sampling, minimum and maximum variation sampling strategies were used to ensure a wide range of perspectives [17]. The participants were recruited across Germany from all federal states via Facebook, through the author’s personal contacts and home care services. None of the participants worked at the same home care service. The providers of the home care services were equally divided between private providers and the non-statutory welfare services. They were located in rural settings as well as in urban settings. The interviewees were between 25 and 62 years old (mean: 38 years, median: 36 years) and had an average work experience of 17 years (min: 5 years, max: 43 years, median: 12 years).

With the consent of the participants, the interviews were recorded, transcribed and anonymized. The interviews lasted on average 76 minutes. They took place at a location chosen by the interviewee, by video calling or telephone. The interview guide addressed the following topics, but was handled flexibly according to the narrative flow of the interviewees:

- participation in the course of disease,
- requirements for participation,
- dealing with uncertainty,
- dealing with limited cooperation and obstacles,
- allocation of roles and responsibilities,
- meaning of normative incentives.

### Data analysis

The data analysis was conducted by content analysis and thematic analysis according to the recommendations of Meuser and Nagel as well as Witzel and Reiter using the MAXQDA software program [17], [18]. First, the interviews were thematically coded and structured. Under each theme the interview segments were coded for a stepwise compression of text. Finally, the codes were generalized to a higher level of abstraction. Via an iterative process, the codes were continuously checked back with the original material. Analytical memos were written continuously to support the analysis. To ensure the quality of data analysis, themes and codes were discussed online in an interpretation group for qualitative research. In particular, the procedure of interpretation was reflected, selected text material was interpreted together and performed interpretations were reviewed. The interpretation group consisted of five members with a social science background. The meetings of the interpretation group were conducted by video telephony. In the following results chapter, quotations from the interviews are presented to illustrate the themes. The brackets after the quotations refer to the participant number and the paragraph number of the interviews.

## Results

### Forms of poverty

Nurses in home care describe different forms of poverty among people in need of care. They differ between (1) an austerity deemed necessary by those in need of care and their relatives, and (2) existent material and financial poverty. 

*“There are also those who, as a war generation, don’t know anything else. It doesn’t matter how much money there is in the account. They’ve internalized saving. […] And with her [patient] there is also the question how long the little money saved will probably last. There’s always her fear: what if she needs more help or has to go to a nursing home?” *(I32:46)

This austerity has often developed due to the biography of the patients, which results in acting daily in fear of future financial distress. In addition, there is the fear of not being able to plan one’s own situation. While patients have always weighed risks in everyday life carefully, they cannot plan or control their new life situation by themselves. In addition to the fear of one’s own increasing physical and mental frailty, there is also often a pronounced concern about how the need for help can be financed going forward or whether one’s own reserves will be sufficient for this. Other patients must deal with objective restrictions. From the nurses’ perspective, however, these people in need of care are affected by a different form of poverty:

*“Well, she [patient] just really has nothing there. 520 euros per month! After the rent’s paid, there’s nearly nothing to live from.” *(I04:09)

This quote illustrates another typical patient situation. It is characterized by severe financial restrictions, such as a scarce pension. In addition, there can be material restrictions, such as little tableware, hardly any laundry and meagre furnishing of their flat. This is particularly critical if the patient has to spend most of the time in his or her home due to their health situation. For example, nurses report situations in which the bed is the only place to sit down or the spartan furnishings are described by the patients themselves as *“comfortable as a prison cell”* (I12:134). Sometimes nurses experience precarious life situations in people in need of care up to the threat of homelessness. These are characterized by debt or a significant lack of basic equipment, e.g. if the sudden occurrence of need for care or a change in life situation due to the death of a relative makes it impossible to cope with outstanding payments:

*“There is a mattress on the floor, no furniture, no kitchen. An ancient kettle and a camping stove were there. The refrigerator hasn’t worked for a long time either.”* (I23:78)

### Strategies in dealing with health and nursing care

The situations described raise the question to what extent nursing care services can provide support if they are involved in the care. Nurses report that people in need of care who are affected by poverty are usually very reluctant to use support services. This is apparent at the first contact:

*“They [patient and her relatives] came in very late. She was already bedridden and also had a pressure ulcer. The atmosphere in the family was tense. But in the initial call the first question was ‘How much does it cost?’ […] Without additional payment, it was only possible to provide the basic package. No matter how much help was necessary from a professional perspective.”* (I04:09)

Due to their financial situation, some families organize necessary care for as long as possible without professional help. If a home care service is consulted, the focus is on selecting one without additional payments. In addition, nurses observe that people in need of care, but also their relatives, are very ashamed to receive financial support (e.g. from the social security office): 

*“She [patient] doesn’t want to be a welfare case. It doesn’t concur with her self-perception. […] And then there’s a lot of paperwork if you make a request to the social security office. Who’s gonna do that? [...].”* (I15:05) 

The high degree of shame and denial of one’s own situation, but also the fear that relatives will be burdened financially, leads to restraint in the use of further support services. This also applies to legitimate claims for supportive social benefits, according to the nurses interviewed. Besides, nurses are often confronted with the concern of those in need of care that the social security office may take control of their life and oblige their relatives to pay. It must also be taken into account that people in need of care and their relatives have often lived in poverty-prone living conditions for years and sometimes perceive their own situation differently (*“others have it even worse”* (I28:177)) or negate it – as an adaptation reaction. Nurses report that, especially in long-term poor living conditions, people in need of care can understand their situation as a *“modest lifestyle”* (I24:314). They identify themselves with this lifestyle with pride and distinguish themselves in this manner from other high-income groups of the population. Against this background, nurses point out that possible support services can usually only be offered in a gradual manner. Another question not to be underestimated is who informs patients about possible social claims at all and who is responsible for the often time-consuming application and administration, since the patients themselves are usually no longer in a position to do so. Here, gaps in the social support of patients and their relatives are described which can hardly be filled at present due to the lack of regular care structures:

*“The woman [patient] is 89 years old; her daughter, who takes care of everything, is 70 years old. They are both unable to cope with such things. She doesn’t have a driver’s license either, no computer. You can’t say ‘take a look in the internet’ or ‘drive 50 km to the next center for social counseling’! But we are nurses in a home care service and cannot counsel about all laws and claims for each individual case.”* (I13:62)

#### Food intake, personal hygiene, living conditions

Another area in which nurses perceive typical patterns in patients affected by poverty coping with their situation is food intake, personal hygiene and living conditions:

*“Well, he [patient] eats just bread with salami or cheese. Sometimes he drinks a clear broth. Sometimes there’s a can. […] Eating something good, it doesn’t matter anymore […]. An unhealthy one-sidedness.”* (I04:09)

Particularly characteristic for such patients in need of care is the restriction of the choice and intake of food, so that the nurses fear malnutrition or also hygienically precarious conditions (e.g. when there is a lack of food cooling). Also described is the renunciation of body care products and showers or bathing, as well as the lack of heating of living spaces in order to save money. Old clothes and household linen also distress nurses in their care work:

*“Three washcloths, two towels, two bedding sets, no laundry dryer. That’s all she’s [patient] got. And she is incontinent. But where do you get the necessary things from? […]”* (I15:05) 

When nurses observe such deficiencies, they sometimes try to compensate the situation by donating things from their own household, for example towels, but also bring home-cooked food or even cheap clothing for those in need of care. Support through other professional social structures is only described in single cases. Some interviewees point out the lack of projects or complain about the project status itself, which only offers temporary help: 

*“As soon as we hear about the project, it’s over and there’s no more help”* (I04:198). 

Low-threshold support services, which are typically often set up as time-limited projects, also make it difficult for nursing services to obtain information about possible support options for people in need of care and to build up functioning networks that can be accessed if necessary. At the same time, the nursing staff state that such deficiency situations are rarely addressed by the patients themselves. The situation is different, however, when it comes to social activities:

*“Sometimes she [patient] cries […] because she’d like to go to the café. She’s very lonely. She doesn’t see anyone except us [home care service]. But it costs four or five euros each time and she doesn’t have the money.”* (I29:25)

Many interviewees describe the absence of social activities such as senior citizens’ cafés or reading magazines as necessary so that the monthly budget of those in need of care is not exceeded. However, people in need of care perceive this as particularly stressful and as a restriction of their quality of life. To compensate for this situation, some patients affected by poverty “recycle” discarded items if they are physically able to do so, e.g. by taking newspapers from their neighbor’s waste and reading them.

#### Dealing with one’s own health

In dealing with health, nurses describe characteristic situations that can be found in patients whose living conditions are marked by poverty. This includes hygienically precarious handling of wound material and incontinence articles, as described in the following quotation:

*“[…] she [patient] dries the incontinence pants and reuses them. This saves co-payments. Of course, this is not hygienic, and it smells.”* (I04:09)

But there are also strategies for dealing with medication that point to inappropriate handling:

*“[…] when the eye drop bottle is half empty, she [patient] fills it up with water. Works just as well and costs half as much, she says.”* (I15:05) 

In other cases, nurses describe how disinfectants are diluted or how doctor’s visits are reduced to save travel costs. Another strategy described is where patients sometimes change their medication intake, e.g. daily tablets for high blood pressure are taken only every other day, or spouses share the patient’s medication. Thereby, patients aim for a reduction in costs by lowering consumption. The nurses interviewed report that many patients and their relatives are not sufficiently informed about the possibility of co-payment exemption or there are diffuse concerns regarding financial social support services as described above. For aids and medicines that are generally not reimbursable by the health insurance, patients in need of care often find *“their own creative, sometimes dangerous solutions”* (I2278):

*“Actually, he [patient] should get new glasses […]. He can’t see properly, and his own glasses are fixed with duct tape. But he didn’t get the money for new ones.”* (I31:21) 

Aids such as glasses, hearing aids and dentures are often not purchased for financial reasons. The nurses interviewed report that the lack of adequate aids is an additional hindrance in communicating with those affected, especially when age-typical impairments of vision and hearing or major damage to dentures occur concurrently.

### Consequences for participation opportunities

As the previous results suggest, the strategies of those in need of care and affected by poverty have consequences for their opportunities for participation. The nurses interviewed reported three areas that are particularly characteristic. Firstly, affected patients appear to be particularly limited in the use of home nursing services, as the avoidance of co-payments is more important than their existing need. Secondly, the participation in social contact is limited. Thirdly, access to medical care is also restricted if, for example, medication and travel costs become an obstacle for patients. Otherwise, there are unlimited opportunities for participation in the care situation, for example regarding the visiting time of the home care service or the order of care activities. Consequently, for people affected by one of the forms of poverty described, only a reduced version of participation remains. This means that their opportunities for participation extend only to a very narrowly defined area of their health and nursing care.

## Discussion

Overall, the results show that the potential consequences of poverty in old age affect the everyday life of those in need of care. In some cases, the self-restriction and limited opportunities for participation can be harmful, e.g. through the multiple use of medical aids, but also if the food intake is restricted. Restrictions in social activities especially can be a considerable burden on the quality of life of those affected. This confirms the findings of other studies, which show considerable restrictions in participation by those in need of care who are affected by poverty [12], [14], [15], [19]. Furthermore, the results are also of considerable importance for the medical treatment of the affected patients. The described strategies of patients can make therapies ineffective, affect the quality of life and significantly risk the medical safety of patients. In practice, however, these underlying circumstances may not always become apparent for physicians and nurses or are mistakenly classified as compliance problems. For example, the interviewed nurses described their own lack of knowledge about how to systematically identify patients affected by poverty. This points to a possible need for future research to quantitatively verify this assumption and, if necessary, to develop approaches that prepare health professions for dealing with such patient groups.

In addition, the results suggest that the incalculable costs of care can lead to considerable preventive self-restriction. The main reasons are the unpredictable progress of one’s health as well as the lack of transparency of care costs. While in other areas of life predictive models estimate expected costs for individuals (e.g. when buying property), there is a lack of comparable assistance in the care sector. Furthermore, complexity in the use of support services must be discussed. The social system in Germany provides many opportunities for financial support, but for people in vulnerable life situations these possibilities are nearly incomprehensible, and access to them is highly complex (e.g. responsibility of different insurance systems in the case of need of care). Another group of affected patients are those whose monthly income is insufficient for maintaining basic living standards, for example in the areas of housing and nutrition. Patients’ high degrees of shame and the reinterpretation of their own situation as a lifestyle sometimes lead to negation of their situation. This builds additional barriers to accessing professional support. These results suggest that the number of statistically unreported cases who are actually entitled to social assistance could be significantly higher than the currently supported patients in need of care. At the same time, this may lead to negative selection effects in the use of home care services nursing support and unequal participation opportunities for people in need of care who are affected by poverty [20]. This suggests that social security and health support systems need to be reformed towards a more user-friendly, patient-centred system, which has also been mentioned by other studies [21], [22]. Additionally, the expansion of well-founded, free information, as well as the provision of supportive case management in nationally standardized structures could provide relief for those in need of care.

In addition, the nurses report their lacking knowledge about how to identify supporting professional structures. The interviewees take issue with the fact that social support is often provided by local projects which are limited in time and are constantly changing. In addition, searching, establishing and maintaining social support networks are activities that home care services are not paid for. This means that there is no incentive to include this service in the regular portfolio of nursing services in Germany. The compensation of the situation of nurses as private donors means a de-professionalization of care work and cannot be a solution for the systematic support of the large number of those in need of care who are affected by poverty. Furthermore, the role of general practitioners should also be taken into account. The results of this study suggest that in some cases the problems described are already prevalent before old people in need of care hire a nursing service, e.g. if there are severe financial restrictions, such as a scarce pension. This suggests that general practitioners could be more involved, for example, in systematically establishing early contacts with social support services when older people consult them. However, this requires a strengthening of the link between nursing, medical care and social work in the long-term care sector that should be evaluated in future studies.

The major strength of this study is the detailed insight into the care situation from the point of view of experienced nurses who provide initial information to describe the consequences of poverty in everyday nursing care. It was a helpful approach to gain in-depth results in this hard-to-reach research field. However, the limitations of the results should be considered. Since the study is a secondary analysis, the data were collected for another purpose, which limits the generalizability of the results. Furthermore, the perspective of older people in need for care and affected by poverty, as well as the perspective of their relatives, should also be included in further research to identify the most relevant challenges from their point of view. In addition, in order to plan the adaption of the social system and support structures, a quantification of the problems identified by appropriate study designs will be needed in particular.

Most Western countries have been criticized for not preparing for the complex health needs of their growing ageing populations, which requires new solutions in the long-term management of complex chronic diseases, multimorbidity and associated social demands [23]. The present study points to one of the crucial challenges in this field. Particularly in view of the already high prevalence of people in need of care who are dependent on social benefits, it seems urgently necessary to take a closer look at the situation of those affected through research and also by social policies. This will also require the adaptation of social and health systems to the resulting needs, more low-threshold, interlinked social, medical and care support which has more than a temporary project status, as well as the preparation of the health professions for the associated tasks.

## Notes

### Competing interests

The author declares that she has no competing interests.

### Acknowledgements

The author would like to thank all the participants who gave their time for the interviews.

## References

[1] OECD Health Project (2005). Long-term Care for Older People.

[2] OECD (2011). Help Wanted? Providing and Paying for Long-Term Care.

[3] Eurostat (2017). Population structure and ageing.

[4] Eurostat (2016). People at risk of poverty or social exclusion.

[5] Fontaine R, Pino M, Jean-Baptiste M, Philibert A, Briant N, Joël ME, Börsch-Supan A, Kneip T, Litwin H, Myck M, Weber G (2015). Older adults living with cognitive and mobility-related limitations: social deprivation and forms of care received. Ageing in Europe – Supporting Policies for an Inclusive Society.

[6] Laferrère A, Van den Bosch K, Börsch-Supan A, Kneip T, Litwin H, Myck M, Weber G (2015). Unmet need for long-term care and social exclusion. Ageing in Europe – Supporting Policies for an Inclusive Society.

[7] Lampert T, Hoebel J (2019). Sozioökonomische Unterschiede in der Gesundheit und Pflegebedürftigkeit älterer Menschen. Bundesgesundheitsblatt Gesundheitsforschung Gesundheitsschutz.

[8] Kott K, Statistisches Bundesamt (2018). Armutsgefährdung und materielle Entbehrung. Datenreport 2018: Sozialbericht für Deutschland.

[9] Haitz N (2015). Old-age poverty in OECD Countries and the Issue of Gender Pension Gaps. CESifo DICE Report.

[10] Bundesministerium für Arbeit und Soziales (2017). Lebenslagen in Deutschland. Der Fünfte Armuts- und Reichtumsbericht der Bundesregierung.

[11] Statistisches Bundesamt (2017 [last accessed 2018 Nov 14]). Pflegestatistik 2015. Pflege im Rahmen der Pflegeversicherung. Deutschlandergebnisse.

[12] Falk K, Heusinger J, Kammerer K, Khan-Zvornicanin M, Kümpers S, Zander M (2011). Arm, alt, pflegebedürftig: Selbstbestimmungs- und Teilhabechancen im benachteiligten Quartier.

[13] Heusinger J, Bauer U, Büscher A (2008). Der Zusammenhang von Milieuzugehörigkeit, Selbstbestimmungschancen und Pflegeorganisation in häuslichen Pflegearrangements älterer Menschen. Soziale Ungleichheit und Pflege.

[14] Heusinger J, Klünder M (2005). „Ich lass mir nicht die Butter vom Brot nehmen!“ – Aushandlungsprozesse in häuslichen Pflegearrangements.

[15] Turcotte PL, Larivière N, Desrosiers J, Voyer P, Champoux N, Carbonneau H, Carrier A, Levasseur M (2015). Participation needs of older adults having disabilities and receiving home care: met needs mainly concern daily activities, while unmet needs mostly involve social activities. BMC Geriatr.

[16] Messer M (2018). Patientenpartizipation aus Sicht der Pflege. Eine Analyse der häuslichen Versorgung von Menschen mit Multimorbidität.

[17] Witzel A, Reiter H (2012). The problem-centred interview. Principles and practice.

[18] Meuser M, Nagel U, Bogner A, Littig B, Menz W (2005). ExpertInneninterviews – vielfach erprobt, wenig bedacht. Ein Beitrag zur qualitativen Methodendiskussion. Das Experteninterview. Theorie, Methode, Anwendung.

[19] Wollesen B, Bischoff L, Stehr G, Niessen J (2016). „Auswirkungen der Armut auf die Gesundheit von Senioren im Bezirk Altona“. Zentrale Ergebnisse des 2. Altonaer Gesundheitsberichts. Gesundheitswesen.

[20] Ku LJ, Liu LF, Wen MJ (2013). Trends and determinants of informal and formal caregiving in the community for disabled elderly people in Taiwan. Arch Gerontol Geriatr.

[21] Berwick DM (2009). What ‘patient-centered’ should mean: confessions of an extremist. Health Aff (Millwood).

[22] WHO (2015). People-centred and integrated health services: an overview of the evidence.

[23] Colombo F, OECD (2015). Health systems are still not prepared for an ageing population. Ageing: Debate the Issues.

